# Pediatric cancer mortality: Analyzing early deaths and fatalities in a resource-limited tertiary care context

**DOI:** 10.1371/journal.pone.0312663

**Published:** 2024-10-28

**Authors:** Ahmed Farrag, Amira Mahmoud Osman, Mohamed Hamdy Ghazaly

**Affiliations:** 1 Pediatric Oncology and Hematological Malignancies Department, South Egypt Cancer Institute, Assiut University, Assiut, Egypt; 2 Division of Pediatric Hematology and Oncology, Department of Pediatrics, Children’s Hospital of Central Switzerland, Lucerne, Switzerland; 3 Department of Pediatrics, Faculty of Medicine, Assiut University, Assiut, Egypt; Columbia University Irving Medical Center, UNITED STATES OF AMERICA

## Abstract

**Introduction:**

Children with suspected cancer may succumb to their bad condition shortly after admission, even before a definitive diagnosis can be reached. We aimed to address the issue of delayed presentation and early deaths among children suspected of having cancer. We analyzed also the types and causes of mortalities across different tumor types.

**Materials and methods:**

A retrospective review of reports from newly admitted patients between 2006 and 2010 at the pediatric oncology department of the South Egypt Cancer Institute (SECI) was done. Parameters included age, gender, diagnosis, symptoms, the interval between initial symptoms and the first visit to SECI, the duration from admission to death, and the cause of death.

**Results:**

Among the 502 patients with confirmed malignancies, 238 (47.4%) succumbed. Causes of death were predominantly treatment-related mortalities (TRM) (66%). Mortalities within hematological malignancies were mainly TRM (81%), whereas solid tumors were primarily disease-related (70%), p <0.0001. The leading causes of TRM was infection (60%).

About 5% of patients experienced early death within 48 hours after presenting to SECI. The mean duration from initial symptoms to the first SECI visit was 67 days, and the period from admission to death averaged 27 hours. Common initial symptoms included abdominal swelling (29.6%), or fever (26%). The leading causes of death were respiratory failure (29.6%), tumor lysis syndrome (22%), or septicemia (22%).

**Conclusions:**

Delayed presentation leading to early deaths poses a significant obstacle to the successful treatment of childhood cancers. Early diagnosis and improved supportive care are essential to enhancing the overall survival, particularly in patients with hematologigical malignancies.

## Introduction

While the overall survival (OS) rate for children with cancer has reached 80% in many high-income countries (HICs) [1], the corresponding figures are significantly lower in lower-middle-income countries (LMICs) and low-income countries (LICs), with rates falling below 30% [2–5]. This disparity in survival rates poses a significant challenge, particularly as children in LICs constitute approximately one-third of the population in these regions. Furthermore, children make up 3–10% of all newly diagnosed cancer patients in these countries, a much higher proportion than the 1% seen in HICs [6, 7].

Unfortunately, approximately 43% of children with cancer worldwide remain undiagnosed, with disparities ranging from 3% in HICs to 57% in LICs and LMICs [8]. It was estimated that a significant number of children with cancer go unnoticed as they succumb to the disease before undergoing diagnosis and registration. Consequently, there are gaps in information concerning the incidence and mortality of cancer [9–13]. Previous studies have noted a higher incidence of childhood cancers in capital cities and metropolitan provinces of LMICs compared to smaller cities. This raises concerns about pediatric patients with malignant tumors in rural or remote areas, who may face challenges in accessing accurate diagnoses and proper therapy [14].

Factors such as the lack of tertiary healthcare centers in rural areas, parental illiteracy, and limited health-related knowledge contribute to delays in seeking medical advice for affected children. Traditional healers are often consulted, and access to comprehensive treatment remains limited [10, 15, 16].

The lack of awareness about cancer, its symptoms, and its signs in children is not just within the general community but also among healthcare members in many rural areas. Patients may need several visits to different healthcare providers before an accurate diagnosis can be established and effective therapy can be started. This prolonged period of undiagnosed illness leads to delayed referrals to specialized centers, resulting in advanced-stage presentations at cancer centers [10, 17]. For many patients, this delay translates into palliative care and pain management as the only viable options [12]. Factors contributing to delayed diagnoses include the overall rarity of childhood cancer, non-specific disease manifestations, unavailability of specialized physicians in primary care units, and patients’ reluctance to seek medical advice, often due to the misconception that cancer is generally incurable [7, 18]. Early diagnosis is crucial for improving survival rates, and some studies indicate that certain delayed cancer diagnoses are preventable [19, 20].

While distinguishing between treatment-related mortality (TRM) and disease-related mortality (DRM) (either disease progress or relapse) may pose challenges [21], it is crucial to identify the dominant factor to enhance OS effectively. If TRM is the primary contributor, interventions should focus on improving supportive care and reducing therapy intensity [22].

We aimed to evaluate the issue of delayed presentation and early deaths among suspected pediatric cancer patients. Additionally, we seek to analyze the types and causes of mortalities within a pediatric oncology department situated outside the capital in an LMIC.

## Materials and methods

### Study design

Significant differences in survival rates among pediatric oncology patients between a center in Egypt and one in Germany were highlighted in a previous study [5]. That study identified early deaths and TRM as major challenges at SECI, but these issues were not further analyzed. Therefore, we conducted the current study to explore these two specific problems in greater details. This retrospective cohort study was conducted in the pediatric oncology department at the South Egypt Cancer Institute (SECI), Assiut University, Egypt. A comprehensive review of available data, both electronic and paper-based records/reports, was performed retrospectively. The collected data included age, gender, diagnosis, duration from the patient’s initial symptoms to the first visit to SECI, date and time of the first visit, initial symptoms, travel time duration from the patient’s home to SECI (estimated using Google Maps in hours), and if applicable, the date, time, and causes of death.

All patients, regardless of gender, up to 18 years old, who were referred to the pediatric oncology department at SECI with a suspected malignant disease between January 1, 2006, and December 31, 2010, were included. Exclusions comprised patients older than 18 at the time of diagnosis, those with a non-malignant disease or a relapsing malignant disease at the first presentation, individuals who had received prior chemotherapy outside SECI, referrals to other hospitals before initiating treatment, patients abandoning therapy before commencing treatment, and those lacking available data.

In this study, early death was defined as death within 48 hours after first admission to SECI. Mortality types were categorized as TRM or DRM, which included two subtypes: Disease-Progression-Related Mortality (PRM) and Disease-Relapse-Related Mortality (RRM). Tumor lysis syndrome (TLS) was considered a TRM if therapy had been initiated. PRM referred to death due to disease progression before achieving complete remission, while RRM denoted death resulting from disease relapse and progression post prior attainment of complete remission. Deaths from unknown causes during treatment, or in cases where it was difficult to determine whether the cause was TRM or DRM, were classified as other causes of death.

This study comprised two main parts. The first focused on the issue of delayed presentation and early deaths within the cohort of patients with either confirmed or suspected cancer, including those who died before obtaining a final diagnosis. The second part aimed to evaluate the types and causes of mortalities, specifically disease-specific mortality causes, in all patients with confirmed cancer diagnoses who were referred to SECI and did not abandon therapy. Abandoned patients usually have a known fate.

Ethical approval was obtained from the scientific research ethics committee at South Egypt Cancer Institute. Informed consent was waived by the ethics committee and considered unnecessary due to the nature of the study. All data were analyzed anonymously.

### Statistical analysis

Data were analyzed in the period from June 15, 2023, to December 31, 2023. Authors had access to information that could identify individual participants during data collection. Data analysis was performed using "Statistical Package for Social Science" (SPSS) version 22. Descriptive statistics were done, such as number, percentage, mean, and median. Group comparisons for categorical data were conducted using the chi-square test, while unpaired t-tests were used for continuous data. Survival groups were estimated using the Kaplan-Meier method.

## Results

**Fig 1:**
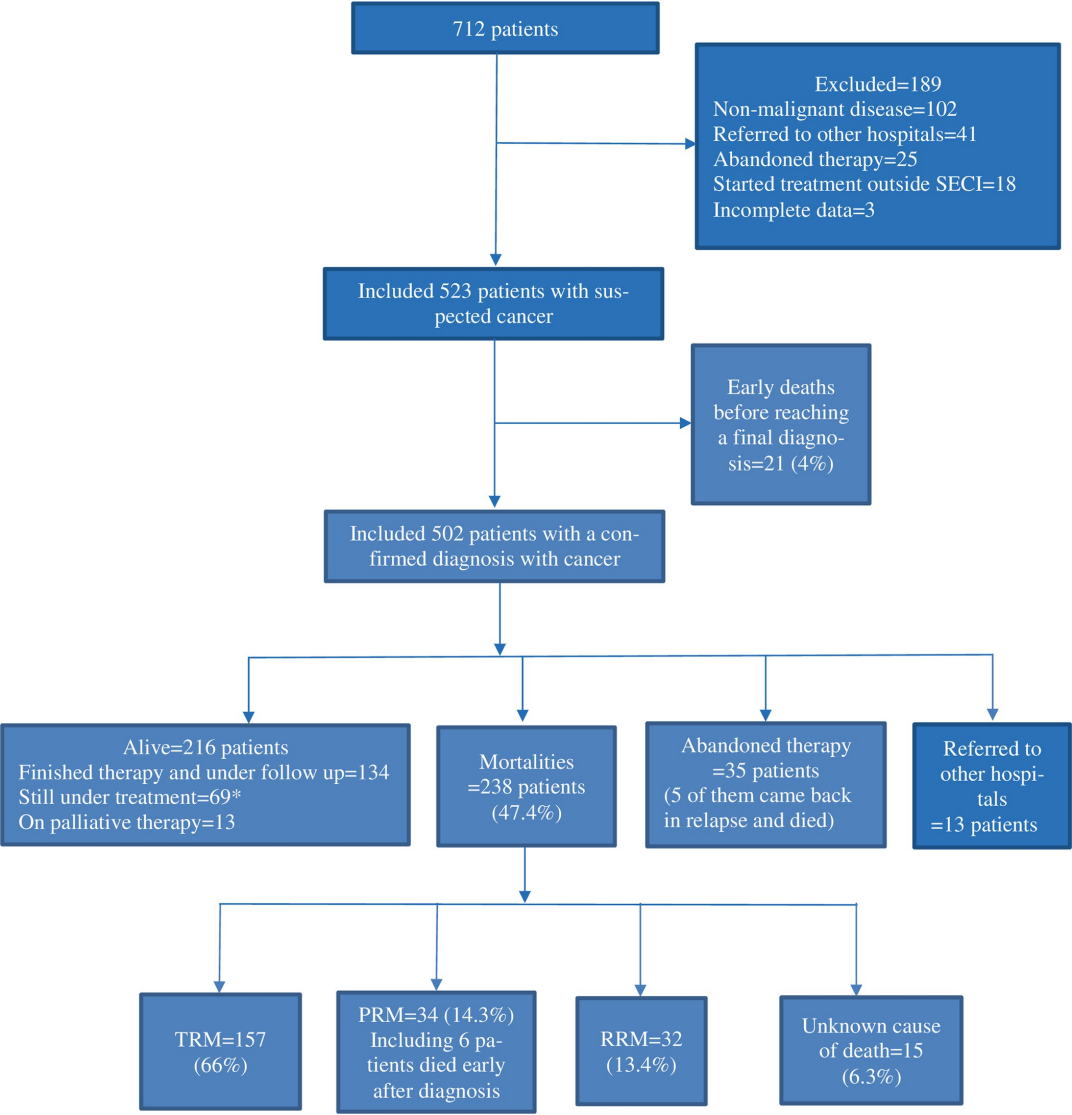
Mortalities among patients first presented between 2006 and 2010 to the pediatric oncology department at South Egypt Cancer Institute (SECI).

Out of a total of 712 children (415 males and 297 females, 1.4:1 ratio) newly presented as suspected pediatric oncology patients at the Pediatric Oncology Department, SECI, Assiut University, Egypt, during the period from January 1, 2006, to December 31, 2010, 523 patients (73.5%) were included in this study. Among them, 502 patients received a confirmed diagnosis of cancer, while tragically, 21 patients with a high suspicion of cancer died early (within 48 hours) after their initial presentation to SECI, before reaching a final diagnosis (Fig 1).

### Early deaths

A total of 27 patients (5.2%), comprising 17 males and 10 females (1.7:1 ratio), with a median age of 8 years (ranging from 0.42 to 15 years), succumbed within 48 hours after their presentation to the pediatric oncology department at SECI. The mean duration from the onset of initial symptoms to the first visit to SECI was 67 days, and from admission to death was 27 hours. Among them, 6 patients had a confirmed diagnosis of malignancy, while the remaining 21 patients had a high suspicion of cancer but died before a final diagnosis could be made. All parents declined autopsy.

The six patients diagnosed with malignancy were predominantly male (n = 4) and predominantly presented with acute leukemia (n = 5), with four of them having acute lymphoblastic leukemia (ALL) in the high-risk group and one with acute myeloid leukemia of unknown risk. Additionally, one patient suffered from a solid tumor (rhabdomyosarcoma) of unknown stage. The median travel duration from their homes to SECI was 0.85 hours (ranging from 0.3 to 6.8 hours), and the median duration from initial symptoms to the first presentation at SECI was 2 weeks (ranging from 1 to 16 weeks), and the mean time till death was 21 hours. Chemotherapy could be started in just one patient, while the others succumbed before the commencement of any anti-cancer treatment, receiving only supportive care.

The diagnostic delays were notable in several cases. Two patients with evident leukemia symptoms (bone pain, fever, pallor, purpura, and ecchymosis) experienced delayed diagnosis (4 weeks in one patient and 16 weeks in the other). Another patient with severe bone pain and headache faced misdiagnosis for 2 weeks before a blood picture revealed hyperleukocytosis with peripheral blasts. One patient with abdominal swelling and recurrent urine retention for 3 weeks encountered a delay in pathology diagnosis, which ultimately revealed rhabdomyosarcoma. Additionally, one patient with a non-specific headache for a week and another with progressively enlarging cervical swelling for one week died early. Both patients had a diagnosis of ALL, which was confirmed after a blood picture revealed peripheral blasts. Three of these five patients with acute leukemia died of sepsis, and two succumbed due to the tumor burden (mediastinal syndrome or TLS) (Table 1). We could not find a significant difference in the examined possible risk factors between patients who died early and those who did not (Table 2).

**Table 1:** Early mortalities within pediatric oncology patients first presented between 2006 and 2010 to SECI.

	Age (years)	Gender	Diagnosis	Risk group	Travel time to SECI (hours)	Duration from initial symptoms to the first presentation of SECI (weeks)	Initial symptoms*	Chemotherapy	Main causes of death	Days of OS	Other information	Delayed	A preventable cause of death
1	9	M	ALL	HR	1.35	4	Bone pain, pallor, purpura	None	Sepsis	1	anemic heart failure, bronchopneumonia	Yes	Yes
2	11	M	ALL	HR	0.3	1	Headache	None	Sepsis	0	Initial TLC 450 X10^3^/μL, Bronchopneumonia	No	Yes
3	12	M	ALL	HR	0.85	1	Cervical swelling	None	mediastinal syndrome	1	Septic arthritis	No	No
4	13	M	ALL	HR	0.7	2	Bone pain, headache	None	TLS	2	Initial TLC 950 X10^3^/μL, CNS positive, convulsions	Yes	Yes
5	3	F	AML	unkwon	3.2	16	Fever, pallor, ecchymosis	Started	Sepsis	2	Fever neutropenia	Yes	Yes
6	3	F	RMS	unknown	6.8	3	Urine retention, abdominal distention	None	DCL, irregular breath	2	Initial Hb 6	Yes	No

SECI indicates South Egypt Cancer Institute; OS, overall survival; M, male; F, female; ALL, acute lymphoblastic leukemia; AML, acute myeloid leukemia; RMS, rhabdomyosarcoma; HR, high risk; TLS, tumor lysis syndrome; DCL, disturbed conscious level; TLC, total leucocytic count; CNS, central nervous system; and HB, hemoglobin.

*Main symptom mentioned initially.

**Table 2:** Risk factors affecting occurrence of early deaths of newly diagnosed and treated pediatric oncology patients between 2006–2010 in SECI.

Risk factor	Early deaths	Non-early deaths	p-value
Age (years) Mean ± SEM	8.5 ± 1.8	6.7 ± 0.29	0.3087
Gender			
Male	4 (66.7%)	122 (52.6%)	0.6868
Female	2 (33.3%)	110 (47.4%)	
Travel duration (hours) Mean ± SEM	2.2 ± 1.0	1.4 ± 0.1	0.2124
Duration of symptoms (weeks) Mean ± SEM	4.5 ± 2.4	5.8 ± 0.5	0.6779
Type of disease			
Hematological malignancies	5 (83.3%)	180 (77.6%)	1.000
Solid tumors	1 (16.7%)	52 (22.4%)	
Risk group*			
HR	4 (66.7%)	125 (53.9%)	0.5737
Non-HR	0 (0%)	40 (17.2%)	

SEM indicates standard error of mean.

*69 patients (two within early deaths and 67 within non-eary deaths) had an unknown stage/risk stratification.

Twenty-one patients (4%) (13 males and 8 females, 1.6:1), median age 3 (0.5–15) years, presented in a poor general condition and died early (mean 29 hours) after presentation to the Pediatric Oncology Department and before reaching a final diagnosis or starting any anti-cancer therapy. These patients had a high suspicion of cancer and, therefore, were referred to SECI. The median travel time from their homes to the cancer center was 1.35 hours (range 0.3–3.15 hours). They had a median duration of 4 weeks (10 days-96 weeks) from the onset of symptoms till presenting to SECI (3 (1–96) weeks in females and 4 (1–16) weeks in males). The main complaints of these patients were abdominal swelling (n = 7, 33.3%), fever (n = 6, 28.6%), head and neck swellings (n = 3, 14.3%), bleeding (n = 3, 14.3%), or pallor (n = 2, 9.5%). Most of these patients succumbed within minutes to 24 hours after presentation to SECI (n = 15). Causes of death included respiratory failure (n = 8, 38.1%), TLS and acute renal failure (n = 5, 23.8%), septicemia (n = 3, 14.3%), mediastinal syndrome (n = 2, 9.5%), anemic heart failure (n = 2, 9.5%) and one patient (4.8%) with status epilepticus and suspected intracranial hemorrhage.

### Mortalities in children with cancer

Five hundred two patients (291 males and 211 females, 1.4:1) with a confirmed diagnosis of malignancy received therapy in the Pediatric Oncology Department at SECI during the study period. The median age at first presentation was 5.2 years (range from 2 months to 18 years). Common diagnoses included leukemia (48.8%), lymphomas (24.1%), solid tumors (24.7%), and rarely brain tumors (1%). The 5-year OS was 45%, and the 5-years EFS was 36%. Tragically, 238 patients (47.4%) succumbed, with two of them dying at home or on their way from a distant home to the hospital. Causes of death were attributed to treatment-related factors in 157 patients or to disease-related factors (tumor relapse/progression) in 66 patients. The cause of death remained unknown or difficult to determine (tumor or toxicity-related) in 6.3% (n = 15) of the deceased patients [5].

A comparison of leading causes of death between hematological malignancies and solid tumors revealed that mortalities from hematological malignancies were predominantly TRM (81% vs. 30% in solid tumors). Conversely, mortalities within patients with solid tumors were mainly DRM (70% vs. 19% in hematological malignancies) p<0.0001.

Treatment-related mortality was the primary cause of death in 157 patients (66% of all causes of death), 82 males and 75 females, median age of 6 (0.4–18) years, and mean of 6.85±0.35 years. Main causes were related to infection (n = 143, 60%), hemorrhage and unavailability of fresh blood products (n = 17, 7.1%), late presentation with huge tumor burden resulting in TLS (n = 13, 5.5%), chemotherapy-toxicity (n = 10, 4.2%), anemic heart failure in patients presented in a bad general condition and unavailability of blood products (n = 9, 3.8%), or acute renal failure (n = 2, 0.8%). In some cases, combined causes occurred, e.g., infection and hemorrhage. Deaths due to other than TRM causes occurred in 81 patients, 37 males, and 44 females, with a median age 5 (0.4–17) years, and mean of 6.46 ±0.48 years.

Mortalities within patients with hematological malignancies (n = 185, 49.6%) were comparable to mortalities within patients with solid tumors (n = 53, 41%), p = 0.1024. However, the distribution of causes of death within these groups showed significant differences (p = 0.0001). Mortalities within hematological malignancies were primarily attributed to TRM (n = 143, 81%), whereas in solid tumors, DRM were more prevalent (n = 33, 70%) (Tables 3 and 4).

**Table 3:** Risk factors affecting the type of death of newly diagnosed and treated patients between 2006–2010 in SECI.

	TRM	DRM	p-value
Age (years) Mean ± SEM	6.85 ± 0.4	6.57 ± 0.5	0.6618
Gender			
Male	82 (52.2%)	34 (51.5%)	1.0000
Female	75 (47.8%)	32 (48.5%)	
Type of disease*			
Hematological malignancies	143 (91.1%)	33 (50%)	0.0001
Solid tumors	14 (8.9%)	33 (50%)	
Risk group**			
HR	84 (53.5%)	38 (57.6%)	0.5487
Non HR	23 (14.6%)	14 (21.2%)	

SECI indicates South Egypt Cancer Institute; TRM, therapy-related mortality; DRM, disease-related mortality; SEM, standard error of the mean; and HR, high-risk.

*9 patients with a hematological malignancy and 6 patients with solid tumors had an unknown cause of death.

**44 patients (30 within TRM and 14 within DRM) had an unknown stage/risk stratification.

**Table 4:** Leading causes of death per diagnosis of newly diagnosed and treated patients between 2006–2010 in SECI.

Diagnosis	Number of patients	All mortalities	TRM	RRM	PRM	Mortalities from other causes
ALL	186	99 (53.2%)	72 (38.7%)	18 (9.7%)	2 (1.1%)	7 (3.8%)
AML	58	50 (86.2%)	44 (75.9%)	2 (3.4%)	4 (6.9%)	0 (0%)
CML	1	1 (100%)	0 (0%)	1 (100%)	0 (0%)	0 (0%)
Brain tumors	5	3 (60%)	0 (0%)	0 (0%)	0 (0%)	3 (60%)
NHL	80	33 (41.3%)	26 (32.5%)	3 (3.8%)	2 (2.5%)	2 (2.5%)
HL	41	0 (0%)	0 (0%)	0 (0%)	0 (0%)	0 (0%)
Neuroblastoma	41	22 (53.7%)	7 (17.1%)	4 (9.8%)	10* (24.4%)	1 (2.4%)
Wilms’ tumor	29	6 (20.7%)	2 (6.9%)	3 (10.3%)	1 (3.4%)	0 (0%)
Osteosarcoma	6	4 (66.7%)	1 (16.7%)	0 (0%)	2 (33.3%)	1 (16.7%)
Ewings sarcoma	3	1 (33.3%)	0 (0%)	0 (0%)	1 (33.3%)	0 (0%)
GCT	9	1 (11.1%)	0 (0%)	1 (11.1%)	0 (0%)	0 (0%)
RMS	11	5 (45.5%)	1 (9.1%)	0 (0%)	3 (27.3%)	1 (9.1%)
Hepatoblastoma	6	3 (50%)	2** (33.3%)	0 (0%)	1 (16.7%)	0 (0%)
Retinoblastoma	4	0 (0%)	0 (0%)	0 (0%)	0 (0%)	0 (0%)
PNET	6	4 (66.7%)	1 (16.7%)	0 (0%)	3 (50%)	0 (0%)
Other sarcoma	3	2 (66.7%)	0 (0%)	0 (0%)	2 (66.7%)	0 (0%)
Other carcinoma	6	2 (33.3%)	0 (0%)	0 (0%)	2 (33.3%)	0 (0%)
LCH	3	1 (33.3%)	1 (33.3%)	0 (0%)	0 (0%)	0 (0%)
MDS	4	1 (25%)	0 (0%)	0 (0%)	1 (25%)	0 (0%)
Total	502	238 (47.4%)	157 (31.3%)	32 (6.4%)	34 (6.8%)	15 (3%)

SECI indicates South Egypt Cancer Institute; TRM, therapy-related mortality; RRM, relapse-related mortality; PRM, progress-related mortality; ALL, acute lymphoblastic leukemia; AML, acute myeloid leukemia; CML, chronic myeloid leukemia; NHL, non-Hodgkin lymphoma; HL, Hodgkin lymphoma; GCT, germ cell tumor; RMS, rhabdomyosarcoma; PNET, primitive neuroectodermal tumor; LCH, Langerhans cell histiocytosis; and MDS, myelodysplastic syndrome.

* 9 of them stage 4 and one stage 3.

** Both of them died with bronchopneumonia.

Treatment-related mortalities were mainly related to infections, which included septicemia (n = 91), bacterial pneumonia (n = 39), fungal chest infection (n = 1), acute gastroenteritis (n = 3), peritonitis (n = 3), typhlitis (n = 1), meningitis (n = 3), and postoperative septicemia due to burst abdomen and intestinal fistula (n = 2). Hemorrhage occurred in various forms, such as pulmonary (n = 7), intracranial (n = 6), hematemesis (n = 2), or external bleeding (n = 2). Toxicity from methotrexate was observed in 3 patients, while other medications were implicated in toxicity-related deaths in 7 patients. Its is important to note that during the study period, there were instances of shortages in some essential medications, such as antibiotics, as well as limited availability of certain laboratory and radiological facilities. Additionally, there was a shortage of the infusion pumps.

Among hematological malignancies, the most common causes of TRM were infection (91%), bleeding (11.2%), TLS (9.1%), chemotherapy toxicity (7%), and anemic heart failure (6.3%), in contrast, patients with solid tumors showed different proportions, with 92.9% of TRM attributed to infection, 7.1% to bleeding, and no deaths due to TLS. Chemotherapy toxicity was involved in 14.3% of deaths in solid tumor patients. These findings highlight the distinctive patterns of mortality causes between hematological malignancies and solid tumors (Table 5).

**Table 5:** Leading causes of TRM within the different diseases categories in newly diagnosed and treated patients between 2006–2010 in SECI *.

Diagnosis	Total TRM	Infection	Hemorrhage	TLS	Chemotherapy toxicity	Anemic heart failure
ALL	72	64 (88.9%)	7 (9.7%)	8 (11.1%)	9** (12.5%)	6 (8.3%)
AML	44	41 (93.2%)	9 (20.5%)	2 (4.5%)	1 (2.3%)	2 (4.5%)
NHL	26	24 (92.3%)	0 (0%)	3 (11.5%)	0 (0%)	1 (3.8%)
Neuroblastoma	7	7 (100%)	0 (0%)	0 (0%)	1 (14.3%)	0 (0%)
Nephroblastoma	2	2 (100%)	0 (0%)	0 (0%)	0 (0%)	0 (0%)
Osteosarcoma	1	1 (100%)	0 (0%)	0 (0%)	1** (100%)	0 (0%)
RMS	1	1 (100%)	0 (0%)	0 (0%)	0 (0%)	0 (0%)
Hepatoblastoma	2	2 (100%)	0 (0%)	0 (0%)	0 (0%)	0 (0%)
PNET	1	0 (0%)	1 (100%)	0 (0%)	0 (0%)	0 (0%)
LCH	1	1 (100%)	0 (0%)	0 (0%)	0 (0%)	0 (0%)
Others	0	0 (0%)	0 (0%)	0 (0%)	0 (0%)	0 (0%)
Total	157	143 (91.1%)	17 (10.8%)	13 (8.3%)	12*** (7.6%)	9 (5.7%)
Hematological malignancies	143	130 (91%)	16 (11.2%)	13 (9.1%)	10 (7%)**	9 (6.3%)
Solid tumors	14	13 (92.9%)	1 (7.1%)	0 (0%)	2 (14.3%)**	0 (0%)

SECI indicates South Egypt Cancer Institute; TRM, therapy-related mortality; TLS, tumor lysis syndrome; ALL, acute lymphoblastic leukemia; AML, acute myeloid leukemia; NHL, non-Hodgkin lymphoma; RMS, rhabdomyosarcoma; PNET, primitive neuroectodermal tumor; and LCH, Langerhans cell histiocytosis.

*Combined causes are possible.

**include one patients suffered acute renal failure.

*** include two patient suffered acute renal failure.

## Discussion

This study sheds light on the critical issues of delayed presentation and early deaths in a teriary care center located outside the capital in an LMIC. Analyzing the various types and specific causes of mortality in pediatric oncology patients with different diagnoses offers valuable insights into the challenges faced in this setting.

The study’s retrospective nature, limited representation of a single medical center, small sample sizes, and missing data pose limitations. Additionally, the reasons for delayed presentation were not explored in-depth, and the study lacked information on causes for the initial suspicion of cancer in the early death cases. Nevertheless, as the first study of its kind in a pediatric oncology unit outside the capital in an LMIC, it lays the foundation for future comprehensive investigations and highlights areas in need of immediate intervention. Larger, more extensive studies covering the entire country will provide a more representative picture.

A notable finding was that a significant portion (5.2%) of patients initially presented in a severe poor general condition and experienced early deaths within minutes to hours. The refusal of autopsy by parents hindered understanding the precise causes of this presentation. Possible explanations include misdiagnosis by less experienced medical teams in rural areas, lack of facilities for accurate diagnosis, parental illiteracy and poverty leading to delayed seeking of medical advice, dependence on traditional non-medical therapies, which are common in this community [23], or might be related to the aggressive nature of the diseases, or other causes.

Unfortunately, six patients with a confirmed malignancy in this study died early after presentation. The diagnosis could be reached much earlier in most of these cases if just a complete blood picture was done earlier and not after several weeks (in 3 patients with acute leukemia), or if pathology results were earlier available (in one patient). Most of these lives could be saved, as they had preventable causes of death if they were earlier discovered (in 4 patients with sepsis or TLS). Also most causes of death were preventable within the 21 undiagnosed early died patients (respiratory failure, TLS, sepsis, and anemic heart failure, in eight, five, three, and two patients, respectively). As infection is more common than cancer in children, Physicians in special clinics, especially in rural areas, unfortunately oftenly prescribe oral antibiotics for patients who present with infection symptoms. Many abstain from requesting investigations to avoid overwhelming patients with extra costs. They depend only on their clinical experience, which is usually deficient in pediatric oncology. We have previously reported that delayed presentation and occurrence of therapy-related complications were risk factors affecting death in general within this cohort of 502 patients with malignant diseases [5], but we could not find a significant difference in the examined possible risk factors (age, gender, travel duration from home to the cancer center, duration of symptoms, type of disease, or the therapy-related risk groups) between the early died patients and non-early died patients.

The delayed presentation and early deaths observed in this cohort underscore the challenges in managing childhood cancer in this locality. Under-diagnosed and under-reported cases contribute to delayed referral and a lower reported annual incidence of childhood cancers. The reluctance of parents to consent to autopsies further complicates the understanding of these cases. The study points out that early diagnosis could significantly impact outcomes, emphasizing the need for increased awareness in the community and among healthcare workers in rural areas.

A study done in Zambia suggested that only about one out of nine children with malignancy in Zambia presented to the University Teaching Hospital for being correctly diagnosed and treated. The rest of these patients mainly died before reaching the hospital due to the lack of proper initial diagnosis, poor access to primary medical care, poor referral system, and the inability to travel to the tertiary care center [24]. Another study in Uganda showed that the majority of parents had to make many steps of visits to different nodes of health care before reaching the tertiary health care center, i.e., the pediatric oncology unit: 20% of the parents reached the final treatment center at the third node of care, 28% at the forth node, one-third of them at the fifth node, and one-fifth of them at the sixth node or after that (median fifth node of care, range 3–16) [25]. Another study showed that the most considerable difference in incidence rates between LICs and HICs was among leukemia patients. This difference is due to the high prevalence of infectious diseases in LICs, e.g., malaria. The symptoms are usually similar to those of leukemia, e.g., fever and anemia, so many patients with leukemia might die after being misdiagnosed and misreported as an infectious disease [26]. The observed low incidence of brain tumors in LICs may be related to the lack of well-established neuroradiologic as well as neurosurgical resources [27]. Modern diagnostic methods, well-trained pathologists, and pediatric oncologists are also deficient in many LICs and LMICs [6, 10, 14], as they are usually only found in large main cities, depriving many patients in rural areas of reaching them quickly, and leading to long diagnosis delays. The pediatric oncology specialist: patients ratio was 1:1600 in India [28], and Tanzania has only one formally trained pediatric oncologist [29].

The issue of delayed presentation and early deaths may be addressed by increasing awareness about cancer, its symptoms, signs, and potential for cure within the community, as well as among health team workers, particularly in rural areas. Enhancing access to screening and encouraging regular routine visits to pediatricians for health check-ups may also lead to earlier referrals to cancer centers.

The observed gender ratio in patients with delayed presentation and early deaths (1.7:1) did not show a significant difference compared to other patients presenting for diagnosis and therapy (1.4:1), p = 0.6910. This contradicts the historical trend of gender preferences in many LMICs [30].

Several previous studies showed that mortalities in patients with hematological malignancies were predominantly due to TRM [31–33]. In this study, TRM in hematological malignancies was the main cause of death, accounting for 76%, 39%, and 32.5% of all patients with acute myeloid leukemia, ALL, and non-Hodgkin lymphoma, respectively. These high TRM rates may be attributed to the restricted facilities for supportive care, as these types of malignancies and their treatments are known for their aggressiveness and the critical need for optimal supportive care. In contrast, TRM among patients with solid tumors was lower. At the time of this study, SECI faced several challenges, including shortages of blood products, some essential medications, diagnostic tools and equipments, such as infusion pumps. These factors likely contributed to the high incidence of TRM.

Deaths in this cohort were primarily therapy-related (66%), with infections accounting for 60% of all causes of death. The second most common cause of TRM was bleeding (n = 17) and anemic heart failure (n = 9), often extracerbated by a lack of blood products, or patient noncompliance and late presentation between the chemotherapy cycles [34]. Our findings at SECI are consistent with those of Saskia Mostert’s studies in Indonesia [35], which also found infection and hemorrhage to be the most common causes of TRM. A previous Study at SECI [5] revealed significant deficiencies in some laboratory and radiological diagnostic capabilities, which slow down efforts to confirm some bacterial, viral, and fungal infections. Additionally, shortage of some essential drugs, including many antimicrobial agents, contributed to delays in identifying the cause of infection and initiating appropriate treatment. A lack of minor equipment, such as infusion pumps, might be responsible for increased chemotherapy-associated toxicity. The high incidence of TLS (n = 13, 5.5% of all deaths) in this cohort is likely due to the fact that many patients presented to SECI at advanced stages of disease, with a significant tumor burden.

Treatment-related mortality are considered preventable causes of death, therefore, a better supportive care or adaptation of the therapy protocols may improve survival in countries with same problems.

Late presentation in late stages with a huge tumor burden or with fulminant infection, massive bleeding, or severe anemia with subsequent early death before reaching a final diagnosis and starting proper therapy are unique problems of childhood cancer management in our locality. Early diagnosis of cancer is an essential aim in oncology, as it facilitates an earlier start of treatment while the tumor is still in its early phase. Better management of treatment-related complications, adapted treatment protocols, and early referral may reduce mortalities and early deaths.

During the preparation of this work the authors used Chat GPT/Open AI in order to improve language and readability, with caution. After using this tool/service, the authors reviewed and edited the content as needed and take full responsibility for the content of the publication.

## Abbreviations key

Abbreviation: Full term
ALL: Acute lymphoblastic leukemia
DRM: Disease-related mortality
HIC: High-income country
LIC: Low-income country
LMIC: Lower-middle-income country
OS: Overall survival
PRM: Disease-Progression-Related Mortality
RRM: Disease-Relapse-Related Mortality
RMS: Rhabdomyosarcoma
SECI: South Egypt Cancer Institute
SEM: Standard error of the mean
SPSS: Statistical Package for Social Science
TLS: Tumor lysis syndrome
TRM: Treatment-related mortality
